# Phosphate of Lime Lozenges

**Published:** 1858-04

**Authors:** 


### PHOSPHATE OF LIME LOZENGES.

Those who wish to prescribe the sub-phosphate of lime, for difficult or imperfect dentition, can obtain it neatly prepared in a pleasant form for administration; they are prepared by H. Packham, Cincinnati, O. Price 25 cents per box, containing 25 Lozenges, each lozenge containing three grs. sub-phosphate of lime.

We have them for sale at the Dental Depot, 38 west 4th Street, Cincinnati, O.

For the value of sub-phosphate of lime in the production and preservation of healthy teeth in children, we would refer to the discussion before the Mississippi Valley Association of Dental Surgeons, at its meeting in February, 1858.

